# Overexpression of miRNA-9 Generates Muscle Hypercontraction Through Translational Repression of Troponin-T in *Drosophila melanogaster* Indirect Flight Muscles

**DOI:** 10.1534/g3.117.300232

**Published:** 2017-09-01

**Authors:** Prasanna Katti, Divesh Thimmaya, Aditi Madan, Upendra Nongthomba

**Affiliations:** Department of Molecular Reproduction, Development and Genetics, Indian Institute of Science, Bangalore 560 012, India

**Keywords:** *Drosophila melanogaster*, Troponin-T, hypercontraction, miRNA, indirect flight muscles

## Abstract

MicroRNAs (miRNAs) are small noncoding endogenous RNAs, typically 21–23 nucleotides long, that regulate gene expression, usually post-transcriptionally, by binding to the 3′-UTR of target mRNA, thus blocking translation. The expression of several miRNAs is significantly altered during cardiac hypertrophy, myocardial ischemia, fibrosis, heart failure, and other cardiac myopathies. Recent studies have implicated miRNA-9 (miR-9) in myocardial hypertrophy. However, a detailed mechanism remains obscure. In this study, we have addressed the roles of miR-9 in muscle development and function using a genetically tractable model system, the indirect flight muscles (IFMs) of *Drosophila melanogaster*. Bioinformatics analysis identified 135 potential miR-9a targets, of which 27 genes were associated with *Drosophila* muscle development. Troponin-T (TnT) was identified as major structural gene target of miR-9a. We show that flies overexpressing miR-9a in the IFMs have abnormal wing position and are flightless. These flies also exhibit a loss of muscle integrity and sarcomeric organization causing an abnormal muscle condition known as “hypercontraction.” Additionally, miR-9a overexpression resulted in the reduction of TnT protein levels while transcript levels were unaffected. Furthermore, muscle abnormalities associated with miR-9a overexpression were completely rescued by overexpression of TnT transgenes which lacked the miR-9a binding site. These findings indicate that miR-9a interacts with the 3′-UTR of the TnT mRNA and downregulates the TnT protein levels by translational repression. The reduction in TnT levels leads to a cooperative downregulation of other thin filament structural proteins. Our findings have implications for understanding the cellular pathophysiology of cardiomyopathies associated with miR-9 overexpression.

Muscle contraction is crucially dependent on the proper assembly, maintenance, and function of myofibrils (Beall and Fyrberg 1991; Gordon *et al.* 2000). Myofibril assembly is a highly complex and coordinated process that requires the maintenance of appropriate stoichiometries of structural proteins and protein complexes, such as the acto-myosin complex and the Tn–Tropomyosin complex (Laing and Nowak 2005; Firdaus *et al.* 2015). Defects, genetically caused or otherwise, in muscle development, structure, or function, result in a number of disorders and diseases, collectively referred to as myopathies (Charge and Rudnicki 2003; Selcen 2011; Gautam *et al.* 2015). Congenital myopathies, including genetic heart diseases, comprise a wide variety of muscle disorders that are mostly due to mutations in the contractile proteins (Chawla 2011; Selcen 2011). For example, mutations of cardiac TnT, α-Tm, and myosin cause hypertrophic cardiomyopathy (HCM) (Watkins *et al.* 1995; Karibe *et al.* 2001). Stoichiometric imbalances of structural proteins and altered isoform expression leading to myocardial damage are also seen in secondary cardiomyopathies, resulting from infection or other factors (Thierfelder *et al.* 1994; Watkins *et al.* 1996; Sisakian 2014). To achieve the appropriate stoichiometric balances, many levels of regulation are required. While transcriptional regulation of these components is extremely important, the significance of the roles of microRNAs (miRNAs) in myopathies in general, and in hypertrophy in particular, are becoming increasingly recognized (Eisenberg and Psaty 2009; Parkes *et al.* 2015).

miRNAs are small noncoding endogenous RNAs, typically 22–30 nucleotide long, that exert subtle control over gene expression, transcriptionally or post-transcriptionally. This makes them an indispensable part of the regulatory network in almost all complex events in organisms ranging from plants to mammals, and even their viruses (Bartel and Chen 2004; Allen and Howell 2010; Carthew and Sontheimer 2009). In muscles, many miRNAs—including both the muscle-specific “myomiRs” such as miR-1, miR-133, and miR-206 (McCarthy 2008), as well as those more widely expressed, such as miR-24, miR-29, and miR-181 (Erriquez *et al.* 2013)—are involved in the regulation of both myoblast proliferation and differentiation (Chen *et al.* 2008). Importantly, the expression of several miRNAs is significantly altered during cardiac hypertrophy, myocardial ischemia, fibrosis, heart failure, and other cardiac myopathies (Latronico and Condorelli 2011; Oliveira-Carvalho *et al.* 2012). miR-9, a miRNA of recognized neural functions (Gladka *et al.* 2012; Krichevsky *et al.* 2003), reportedly plays a regulatory role in myocardial hypertrophy and is antagonistic to myocardin, a positive mediator of cardiac hypertrophy (Wang *et al.* 2010). In addition, miR-9 was implicated in the regulation of platelet-derived growth factor receptor-β, a regulator of cardiomyocyte angiogenesis (Zhang *et al.* 2011). Both studies reported downregulation of miR-9 upon activation of the hypertrophic response. Recently, clinical studies revealed that miR-9 expression levels are significantly lower in hypertensive patients as compared to healthy controls, and appear to be correlated with ventricular mass (Kontaraki *et al.* 2014). Thus, miR-9 appears to have more than one role in cardiac function. It is thus imperative to characterize its roles in the context of muscle development and function. There is high evolutionary conservation of the ultrastructure of striated muscles, their component proteins, and the mechanisms that regulate the assembly of sarcomeres and myofibril formation throughout vertebrates; similarly, there is substantial similarity between the muscles of invertebrates with complex locomotion (Taylor 2006). The *Drosophila* IFMs provide a good system with which to study muscle development, function, and associated diseases (Sparrow *et al.* 2008). This is particularly true with respect to the investigation of cardiomyopathic disorders, as the IFMs exhibit properties such as stretch activation and asynchronous contraction that are physiologically similar to those of cardiac muscles (Vigoreaux 2001). Specific mutations of the *Drosophila* contractile machinery (Kronert *et al.* 1995; Nongthomba *et al.* 2003), signaling cascades (Gajewski *et al.* 2006), and connective tissues (Pronovost *et al.* 2013) lead to muscle hypercontraction, associated with decreased structural integrity of the sarcomeres, which is similar to that seen in many myopathic conditions of higher organisms, including humans. In particular, the *Drosophila* miR-9a is an exact copy of the human miR-9 (Yuva-Aydemir *et al.* 2011).

In this study, we have investigated the regulatory role of miR-9a in *Drosophila* for IFM development and functioning. We show that *Drosophila* miR-9a plays a novel role in the regulation of TnT, a major structural protein, during myofibrillogenesis. This finding will lead to a better understanding of how human miR-9 may be involved in the pathogenesis of cardiac hypertrophy.

## Materials and Methods

### Fly strains and crosses

All flies were maintained on standard cornmeal-agar-yeast medium. Canton-S was used as a control for most of the experiments unless specified. Crosses were performed at 25°, unless otherwise indicated. *UH3-Gal4* (X chromosome) expression from 36 hr after puparium formation (APF) onwards becomes IFM-specific (Singh *et al.* 2014). UAS-miR-9a [third chromosome, Bloomington *Drosophila* Stock Center (BDSC) #41138] and UAS-miR-SP-9a (third chromosome, kind gift from David Van Vactor, Harvard Medical School) were used for overexpression and knocking-down of miR-9a, respectively. UAS-SlsRNAi (third chromosome), UAS-mbcRNAi (third chromosome), and UAS-NeuralizedRNAi (third chromosome) were procured from the BDSC, and UAS-TnTRNAi (third chromosome) was from VDRC, Vienna (v27853). The green fluorescent protein (GFP) construct [sls-GFP (third chromosome)] has been described in Morin *et al.* (2001). All chromosomes and gene symbols are as mentioned in FlyBase (http://flybase.org), unless specifically described.

### Generation of UAS-TnT lacking the miR-9 binding site

Two transgenic fly lines [UAS-TnT (10a) and UAS-TnT (10b)] were generated for the overexpression of either the adult isoform (10a) or the pupal isoform (TnT-10b). The TnT transcripts (both 10a and 10b) were amplified using cDNA extracted from wild-type thoraces, using primers designed to target the 5′- and 3′-UTRs but to exclude the miR-9a binding site. The primers were also modified to incorporate *Eco*RI and *Kpn*I restriction sites for subcloning into the pUAST overexpression vector (TnT FP with *Eco*RI site: 5′-GAACCGCAGAATTCGCTCCTAC-3′, TnT RP with *Kpn*I site: 5′-GTGAAGGAAAGTGGTACCCGAG-3′). The transcripts were cloned into a TA vector and the clones were screened for the presence of 10a or 10b transcripts using reverse primers specific for the alternatively spliced exon (TnT FP + TnT10a RP: 5′-TTGTGCGCTGAGTGAATC-3′ and TnT FP + TnT10b RP: 5′-CGGTGTATTGCTCCTTCT-3′). The presence of the 10a or 10b transcripts in the respective clones was confirmed by sequencing. The sequenced clones and pUAST plasmid were digested with *Eco*RI and *Kpn*I, the released inserts and cut pUAST vector were ligated, and the transgenic constructs were cloned and confirmed by sequencing. The construct, either pUAST-TnT-10a or pUAST-TnT-10b, along with л-helper plasmid (encoding for a transposase) was injected into embryos of white-eyed w^1118^ flies using the Olympus CK-X31- Narishige IM-9B microinjection system. The adult flies (G_0_) that emerged were then crossed with white-eyed flies to produce the F_1_ generation. The transgenic flies were identified by their red eye phenotype.

### Behavioral test

Flight ability was assayed using 2–3-d-old individual flies as described previously (Drummond *et al.* 1991) and the flies were categorized as up-flighted, horizontally-flighted, down-flighted, or flightless. Each fly was flight tested three times.

### Polarized microscopy

For polarized microscopy, 2–3-d-old flies were bisected and processed using a protocol described previously (Nongthomba and Ramachandra 1999). Images were captured using an Olympus SZX12 microscope fitted with an Olympus C -5060 camera.

### Hematoxylin and eosin staining

Hematoxylin and eosin staining of transverse sections of the adult thorax was done as previously described (Pantoja *et al.* 2013). Sections were mounted using Dibutylphthalate Polystyrene Xylene (DPX) mounting medium (Qualigens, Mumbai) and analyzed by light microscopy. Images were acquired using a Leica DFC300FX camera and processed using inbuilt software.

### Confocal microscopy

The bisected fly hemithoraces were processed for immunohistochemical analysis as described previously (Rai *et al.* 2014). Following blocking, samples were treated differently depending on the type of analysis required.

### Scanning electron microscopy (SEM)

SEM analysis of IFMs was used to visualize sarcomeric structure. Three-d-old flies were dehydrated using an alcohol series (50, 70, 80, 90, 95, and 100%). Samples were incubated for 10 min in each dilution with the final dehydration step in 100% alcohol repeated twice. After twice incubating each sample in hexamethyldisilazane for 45 min, they were dried in a desiccator for 24 hr. The head, wings, abdomen, and legs were then removed, transferred to a glass slide, and bisected sagittally. Bisected thoraces were mounted onto an aluminum stub with carbon tape and surface coated by gold sputtering (20 nm thick film) using a Baltec sputter to avoid charging. A Zeiss, Ultra 55, Field Emission SEM with Secondary Electron Detector was used for imaging with an accelerating voltage of 2–5 keV and 8 mm working distance.

### RNA and PCR

RNA was isolated from the IFMs of 2–3-d-old flies. IFMs were removed from the bisected thoraces at 4° and immersed in Trizol (Sigma). Next, RNA was extracted with the help of Trizol (Sigma) as per the manufacturer’s protocol. cDNA was made using 1–2 µg of extracted RNA and a cDNA synthesis kit (Fermentas). The following primers were used: RP49 (FP: 5′-TTCTACCAGCTTCAAGATGAC-3′, RP: -5′-GTGTATTCCGACCACGTTACA-3′); *upheld* (*up*) (FP: 5′-CTCGGGTGTCTCGGGCTCAC-3′ RP: 5′-CTCGAACGAGAAGATCTGGA-3′); and Opa1-like (FP: 5′-AACGGTGGAGCCAGTTCTCG-3′; RP: 5′-TGATCTCCGTCTGCAGCGTC-3′). Quantitative PCR was carried out using DyNAmoTM HS SYBR green mix (F-410L; Thermo Scientific). Fluorescence intensities were recorded and analyzed in an ABI Prism 7900HT sequence detection system (SDS 2.1; Applied Biosystems). The relative changes in gene expression were estimated after normalization to the expression of a housekeeping gene, RP49. For semiquantitative PCR, reactions were set up using the 2× PCR Mastermix (Fermentas) and PCR amplification was carried out using a Mastercycler Nexus (Eppendorf).

### Northern blotting

Detection of miRNA was carried out using the northern blotting protocol of Varallyay *et al.* (2008). RNA was extracted as described earlier, and quantified using a NanoDrop 1000 spectrophotometer (Thermo Scientific). Equal concentrations of samples were loaded on the gel. RNA bands were visualized under a UV transilluminator (JH BIO Innovations Pvt. Ltd.) and transferred onto a Nitrocellulose membrane (Millipore) by semidry transfer. Locked nucleic acid (LNA) Probe for miR-9a (5′-TCATACAGCTAGATAACCAAAGA-3′) and control probe for U6snoRNA (5′-GTCATCCTTGCGCAGGGGCCATGC-3′) was labeled using 1 µl T4 polynucleotide kinase (PNK), 1 µl [γ-^32^P] ATP, and 2 µl PNK buffer, and the final volume was made up to 20 µl. Following incubation at 37° for 60 min, the probe was purified using a Sephadex G-50 column. The membrane was washed twice with 0.5× TBE (Tris Borate-EDTA), allowed to cross-link under UV light for 60 sec, and then incubated at 40° for hybridization with LNA probe in 1× Perfect Hyb Plus buffer (Sigma) for 2–3 hr. The probed membrane was then washed three times with 2× SSC, 0.1% SDS buffer at 40° and exposed to photographic film. The exposed film was scanned using a Phosphor image scanner (GE Typhoon 9500).

### Western blotting

Dissected adult IFMs were homogenized in sample buffer (312.5 mM Tris-HCl pH 6.8, 10% SDS, 0.5% Bromophenol Blue, 50% glycerol, and 25% β-mercaptoethanol) and denatured for 3 min at 95°. Samples were run on a 12.5% resolving gel and western blotting was carried out as described previously (Nongthomba *et al.* 2007). The primary antibodies used to detect specific proteins were: *Drosophila* anti-TnT raised in rat 1:1000 (gift from John Sparrow, UK), *Drosophila* anti-TnI raised in rabbit 1:1000 (gift from Alberto Ferrus, Spain), *Drosophila* anti-Flightin raised in rabbit 1:1000 (gift from Jim O. Vigoreaux, Vermont), *Drosophila* anti-Actin raised in rabbit 1:1000 (gift from John Sparrow, UK), and *Drosophila* anti-α-Tubulin raised in mouse 1:1000 (DHSB).

### Bioinformatics analysis

miR-9a target prediction was done using five different target prediction software suites. These were Miranda (http://www.microrna.org/, Enright *et al.* 2003), Pic-Tar (http://pictar.bio.nyu.edu, Krek *et al.* 2005), the method used by Stark *et al.* (2003) (http://www.russell.embl.de/miRNAs/), EMBL target prediction, and Target scan fly (http://www.targetscan.org/, Lewis *et al.* 2003). All five different algorithms predict miRNA targets, and hence their results should be nonoverlapping. We shortlisted targets recognized by three or more software suitesas significant matches. These shortlisted genes were cross-referenced to a *Drosophila* IFM microarray dataset (https://www.ncbi.nlm.nih.gov/geo/query/acc.cgi?acc=GSE70252) to check for their expression levels in adult IFMs. The miR-9a targets thus obtained were then functionally annotated using DAVID (http://david.abcc.ncifcrf.gov/, Huang *et al.* 2008) and PantherDB software (http://www.pantherdb.org/about.jsp, Mi *et al.* 2005).

### Data availability

We have provided the details of all the web addresses of data resources that we made use of in this study. Other data that support our findings have been included as Supplemental Material and are described in the *Results* section. Supplemental Figure legends are available in File S1.

## Results

### Knockdown of miR-9a in the IFMs during myofibrillogenesis does not affect muscle structure and function

To investigate the role of miR-9a during myofibrillogenesis, we performed IFM-specific knockdown of miR-9a from 36 hr APF. Sarcomeres of the IFMs are established by an organized assembly of their structural proteins between 37 and 46 hr APF (Reedy and Beall 1993; Nongthomba *et al.* 2004). miR-9a is expressed in developing IFMs and the muscle attachment sites (Yatsenko and Shcherbata 2014), and expression is highly reduced in adult IFMs (Supplemental Material, Figure S1A). The knockdown of miR-9a during myofibril assembly had no detrimental effect on flight (Figure S1B). Flies with knockdown of miR-9a (UH3 > miR-SP-9a) exhibited close to normal flight ability, with 74.2% of flies capable of upward flight and 25.8% exhibiting horizontal flight (*n* = 31), which is similar to wild-type, where 80.6% of flies were up-flighted and 19.4% were horizontally-flighted (*n* = 31) (Figure S1B). Further, both wild-type (Figure S1C) and miR-9a knocked-down flies (Figure S1D) had six normal dorsal longitudinal muscles (DLM) fibers in each hemithorax with normal sarcomeric structures (Figure S1, C’–D’’).

### Overexpression of miR-9a in the IFMs during myofibrillogenesis causes hypercontraction

**Figure 1:**
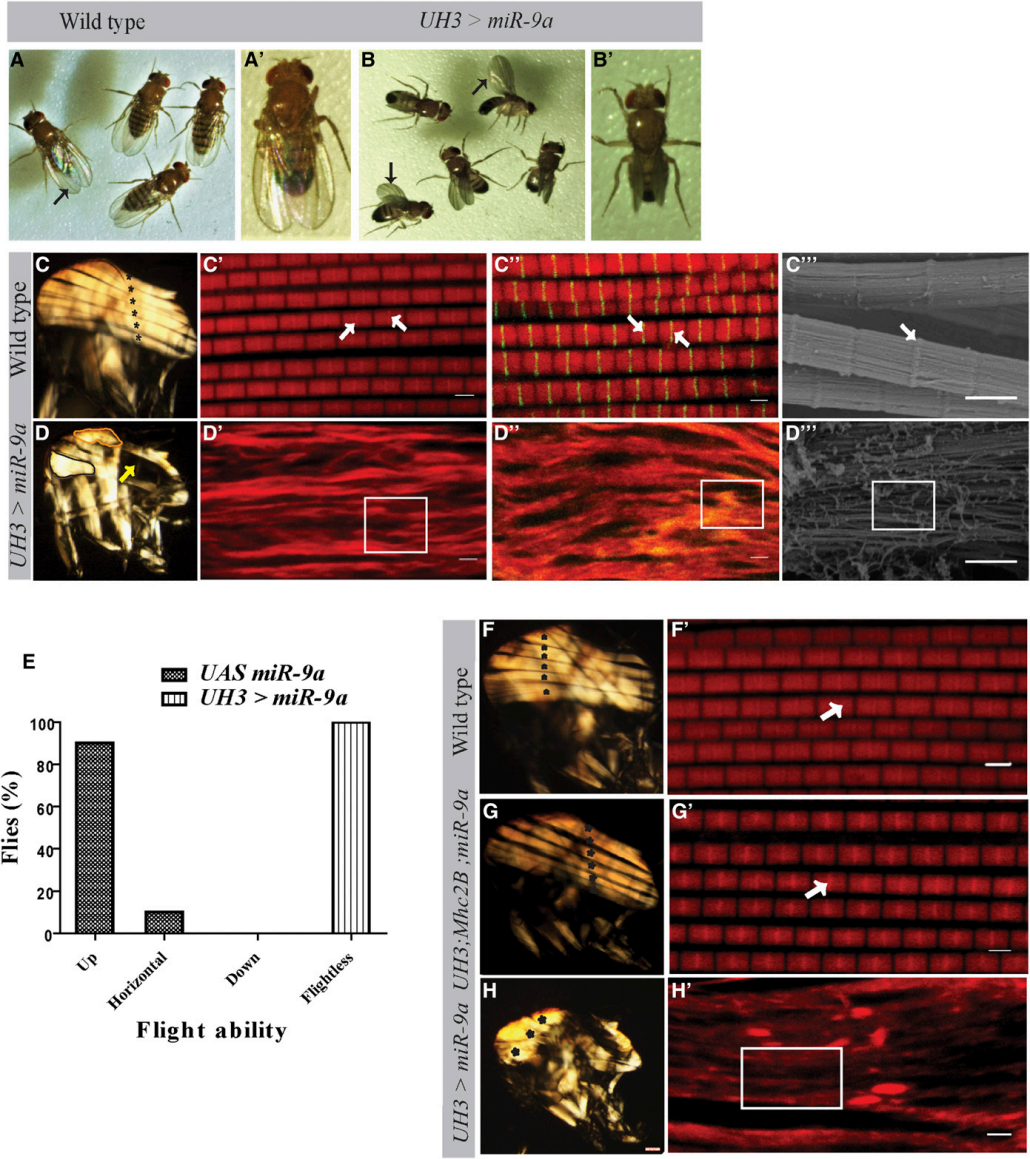
IFM-specific overexpression of miR-9a causes muscle hypercontraction. (A) Wild-type adult flies with normal wing posture. (A’) Regular wing position along the body axis in a wild-type fly. (B) Adult flies overexpressing miR-9a have upheld wings. (B’) Upright wing phenotype in a fly overexpressing mir-9a. (C) Polarized light micrograph of dissected wild-type adult hemithorax showing six DLMs (asterisks). (C’ and C’’) Adult myofibrils stained with Phalloidin-TRITC (F-actin) and Sls-GFP (arrows indicate Z-disc). (C’’’) SEM micrograph of wild-type myofibril (arrow indicates Z-disc). (D) Polarized light image of hemithorax of adult male with overexpression of miR-9a showing broken muscles (arrow) and clumped muscle (outlined in black and orange). (D’ and D’’) Loss of sarcomeric structural integrity (box) in flies overexpressing miR-9a. (D’’’) SEM micrograph showing absence of proper sarcomeres (box) after miR-9a overexpression (bar, 2 µm). (E) Flight assay of the miR-9a overexpression flies. (F) Images of wild-type hemithorax showing six DLMs (asterisks) under polarized light. (F’) Phalloidin-TRITC-stained myofibril showing normal sarcomeric structure (arrow) in wild-type flies. (G) Adult flies carrying a myosin MhcP401S mutation in the overexpression of miR-9a background show six DLMs (asterisks) in the hemithorax and (G’) normal sarcomeres (arrows) in the myofibrils. (H) Polarized light image of hemithorax from flies with overexpression of miR-9a. (H’) Abrogated sarcomeric structure (box) in the myofibrils of flies overexpressing miR-9a (bar, 2 µm). DLMs, dorsal longitudinal muscles; GFP, green fluorescent protein; IFM, indirect flight muscles; miR-9a, microRna-9a; SEM, scanning electron microscope; TRITC, tetramethylrhodamine.

To determine whether the inherently low expression of miR-9a during myofibrillogenesis (Figure S1A) is important for the critical roles of its targets during this stage of muscle development, we overexpressed miR-9a in the IFMs throughout myofibrillogenesis. While miR-9a expression was barely detectable in wild-type IFMs, *UH3-Gal4*-mediated (Singh *et al.* 2014) miR-9a overexpression clearly increased miR-9a levels (Figure S1A) and adversely affected both wing posture and flight performance (Figure 1, A–B’ and E).

The hemithoraces of wild-type flies showed presence of six DLMs (asterisks) with conventional sarcomeric structure, with well demarcated Z-discs (arrows) (Figure 1, C–C’’’). Whereas flies overexpressing miR-9a exhibited broken muscle fibers that appeared to be hypercontracted and pulled toward attachment sites (highlighted in Figure 1, D–D’’’), control wild-type adult hemithoraces showed the typical six well-organized DLM fascicles (Figure 1C) and, at higher magnification, individuals showed well-arranged fibers (Figure 1C’). However, overexpression of miR-9a resulted in abnormal muscle and loss of myofibril integrity in the IFMs, with defects in sarcomeric organization and no organized Z-discs (Figure 1, D–D’’’). Unlike those of the wild-type flies (Figure S2, A and A’), flies overexpressing miR-9a had severe muscle disorganization (Figure S2B), with whole fascicles missing (black arrowheads, Figure S2B’). Flies of both sexes, overexpressing miR-9a, also exhibited a complete loss of flight ability [100% flightless (*n* = 35)] unlike their control counterparts (+; +; UAS miR-9a/+) [90% up-flighted and 10% horizontally-flighted (*n* = 31)] (Figure 1E).

The loss of muscle integrity and sarcomeric structure caused by miR-9a overexpression (Figure 1, D–D’’) is very similar to the hypercontraction phenotype reported earlier (Nongthomba *et al.* 2003). This phenotype is usually associated with mutations in genes encoding sarcomeric structural proteins. It is well established in *Drosophila* IFMs that mutation in some of the structural proteins like TnT, Actin, and TnI can lead a the coordinated reduction in other thin filament proteins. These result in loss of sarcomeric structure and muscle hypercontraction, as a consequence of mis-regulated acto-myosin interaction (Nongthomba *et al.* 2003, 2004, 2007; Firdaus *et al.* 2015). The hypercontraction phenotype can be suppressed in flies with the *Mhc^P401S^* mutation (Figure 1, G and G’) (Nongthomba *et al.* 2003). This mutation is in the Actin binding head region of the myosin heavy chain, which prevents the interaction of the thin and thick filaments, thus reducing muscle contraction. Flies carrying *Mhc^P401S^* mutation in the miR-9a overexpression background showed suppression of the muscle structural defects associated with the miR-9a overexpression (Figure 1, F–H). The DLMs and sarcomeric structure in adult flies (Figure 1, G and G’) were comparable to those of wild-type controls (Figure 1, F and F’). Compared to flies with overexpression of miR-9a alone (Figure 1, H and H’), the hypercontraction suppressed flies possessed six DLMs (asterisks) (Figure 1G) with close to normal sarcomeric structures (Figure 1G’, white arrow shows a Z-disc), confirming that the miR-9a muscle phenotype results from unregulated acto-myosin interactions.

To confirm that the muscle defects observed resulted directly from miR-9a overexpression in the IFMs during myofibrillogenesis, we studied the effect of suppressing miR-9a expression in the overexpression background. Knockdown of miR-9a in the overexpression background rescued the flight ability to levels similar to wild-type flies (Figure S1B). The adult flies had a normal arrangement and pattern of DLMs (Figure S2, D–E’). Thus, the hypercontraction was rescued and the IFMs showed ordered sarcomeres (Figure S2, D–E’) in contrast to those with the phenotype from overexpression of miR-9a alone (Figure 1, D and D’).

### Overexpression of miR-9a results in downregulation of TnT

**Figure 2:**
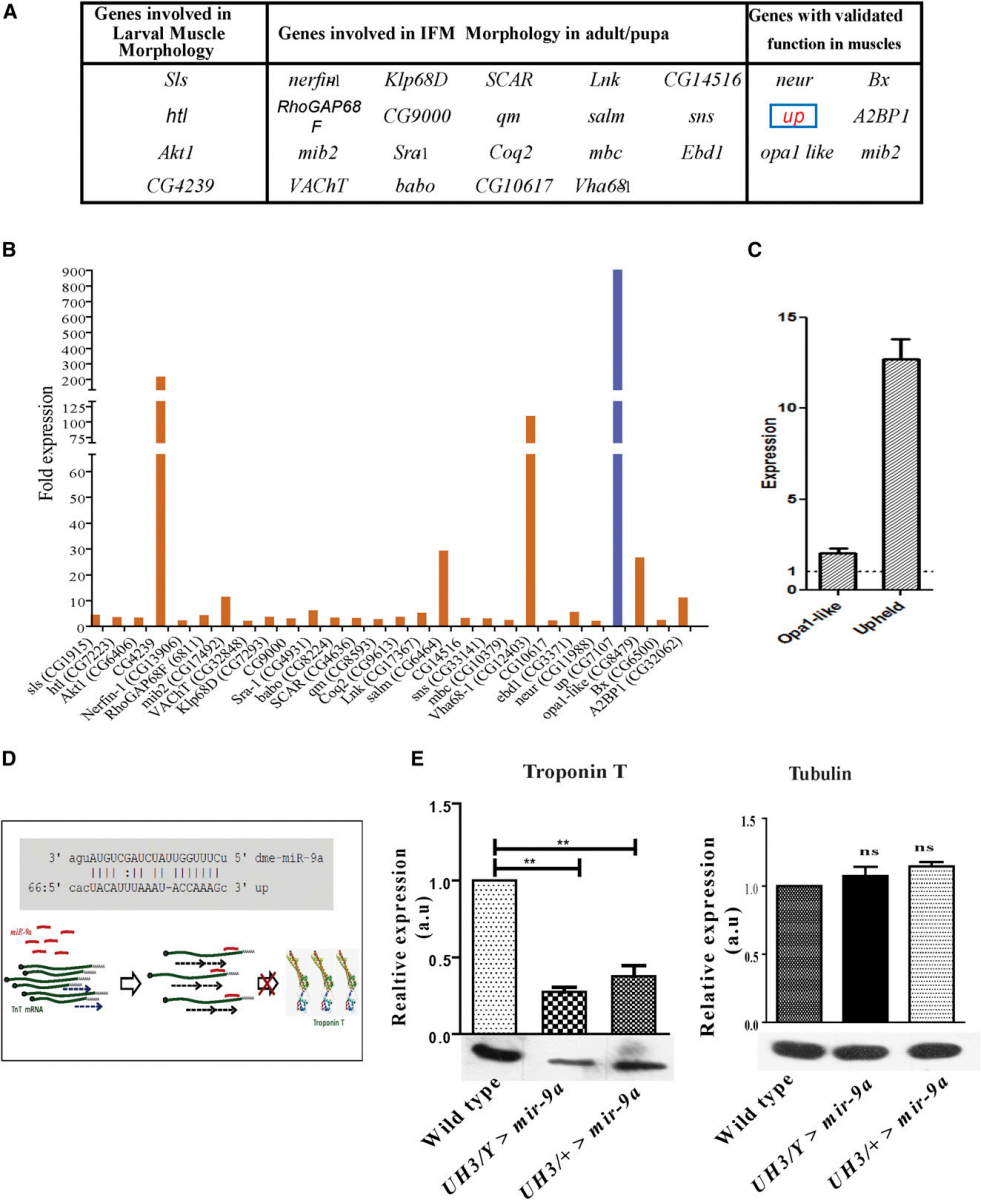
Putative target genes of miR-9a that are involved in muscle development. (A) Lists of genes that are putative targets of miR-9a and their functions in muscles. The *upheld* gene that encodes TnT is highlighted in red (within blue box) (B) Expression profile of the putative miR-9a targets in the IFMs obtained from the microarray data from IFM of wild-type flies. The *upheld* gene showed highest expression in IFM (highlighted by the blue bar). (C) Relative expression of the target genes validated by real-time PCR. (D) Schematic representation of miR-9a binding site at 3′-UTR of *upheld* (TnT) and the mechanism of translation repression of TnT. (E) Quantification of the relative expression of TnT after miR-9a overexpression, using α-Tubulin as loading control (** signifies *P* value < 0.002). IFM, indirect flight muscles; miR-9a, microRna-9a; PCR, polymerase chain reaction; TnT, Troponin-T; UTR, untranslated region; NS, non-significant.

Hypercontraction results from the sarcomeres being unable to withstand the forces produced within them due to changes in the structural proteins of the sarcomere and/or their regulation. Therefore, we investigated if any such proteins are targets of miR-9a. Bioinformatics analysis identified 135 potential miR-9a targets that were then functionally annotated. Five different prediction programs were used (see *Materials and Methods*) and we chose only those predicted targets that were detected by three or more programs. Functional annotation revealed that these genes are involved in a variety of cellular and developmental functions such as transcriptional regulation, protein degradation, apoptosis, endocytosis, neuronal specification, imaginal disc development, etc., but that 29 genes are involved in muscle development (Figure 2A). All the putative miR-9a targets associated with muscle development proved to be involved in muscle function. Of these miR-9a targets in muscles, 4 genes are reported to be involved in larval muscle development, 19 in the development of pupal and adult muscles (Figure 2A) (Schnorrer *et al.* 2010), and 6 genes are associated with muscle development (Madan *et al.* 2017; https://www.ncbi.nlm.nih.gov/geo/query/acc.cgi?acc=GSE70252). These putative targets are expressed in the IFMs as demonstrated using the *Drosophila* IFM microarray (https://www.ncbi.nlm.nih.gov/geo/query/acc.cgi?acc=GSE70252) (Figure 2B).

Among the putative targets of miR-9a genes involved in muscle development and function is the *up* gene, which codes for all the TnT isoforms in *Drosophila melanogaster*. *up* showed a very high level of expression in adult IFMs (blue bar in Figure 2B, expression validated by real-time PCR in Figure 2C). The 3′-UTR of the *up* gene has the miR-9 binding site sequence (Figure 2D). Given that *up* mutations have been implicated in muscle hypercontraction (Nongthomba *et al.* 2003, 2007), we investigated whether miR-9a can cause the downregulation of TnT, possibly through binding to its target sequence at the 3′-UTR of TnT mRNA and suppressing the translational process as represented in Figure 2D. Indeed, there was a significant reduction in TnT protein levels (*P* value < 0.002) in the DLMs of flies overexpressing miR-9a compared to wild-type (Figure 2E). There was also a concomitant decrease in the levels of other structural proteins (which are not targets of miR-9a) that are part of the thin filament (Actin), including the Tn complex (TnI) (Figure S3A). On the other hand, Flightin, a thick filament component, was not reduced in flies overexpressing miR-9a compared to wild-type flies (Figure S3A). These results were expected since reduction of one thin filament protein is known to lead to a coordinated reduction of other thin filament proteins, but does not affect thick filament components (Nongthomba *et al.* 2004, 2007).

### Repression of TnT by miR-9a is responsible for the hypercontraction phenotype

Since several putative miR-9a targets are important during muscle development, we asked whether the hypercontraction phenotype resulted directly from the downregulation of TnT by miR-9a or was due to the repression of other targets. First, we observed that the knockdown of TnT in IFMs during myofibrillogenesis generated a phenotype very similar to the overexpression of miR-9a. Knockdown of TnT resulted in the disruption of muscle structures (Figure S3, E and E’), which was comparable to the muscle defects seen in overexpression of miR-9a (Figure S3, F and F’). We further tested if the knockdown of some other predicted miR-9a targets can lead to similar defects in muscle structure and function. The downregulation of *neuralized*, an E3 ubiquitin ligase, failed to show any muscle defects (Figure S3, C and C’). Flies with a knockdown of *Sallimus* (*Sls*) showed six DLMs (Figure S3D). However, reduction in *Sls*, which is a structural component of the Z-disc, did result in some tearing of the sarcomeres (rectangle, Figure S3D’), but this was not comparable to the damage following miR-9a overexpression.

**Figure 3:**
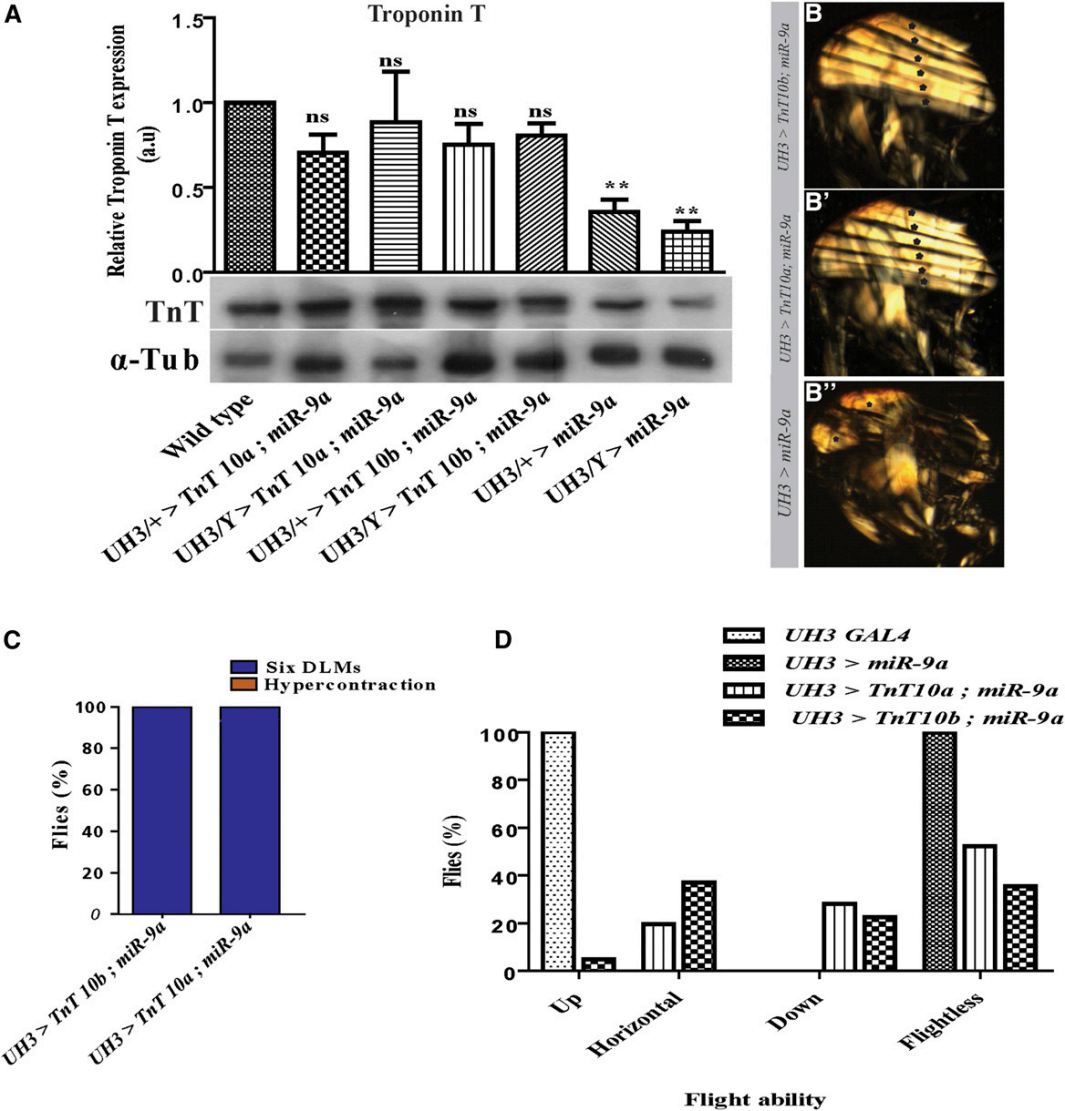
Transgenic lines with overexpression of TnT (10a or 10b isoform) restore Troponin-T levels and rescue the muscle hypercontraction phenotype resulting from overexpression of miR-9a. (A) Western blots and the quantification of the relative expression of Troponin-T (loading control α-Tubulin). (B) Polarized light micrograph showing six normal DLMs following the overexpression of TnT-10a or (B’) TnT-10b isoforms in the background of miR-9a overexpression. (B’’) Polarized image showing hypercontracted muscles after miR-9a overexpression. (C) Quantification of the percentage of flies overexpressing miR-9a that present with hypercontraction phenotype after restoration of TnT levels. (D) Flight data for the flies overexpressing TnT 10a or 10b isoforms in the background of overexpression of miR-9a. ** signifies *P* value < 0.005, DLMs, dorsal longitudinal muscles; miR-9a, microRna-9a; NS, non-significant; PCR, polymerase chain reaction; TnT, Troponin- T.

**Figure 4:**
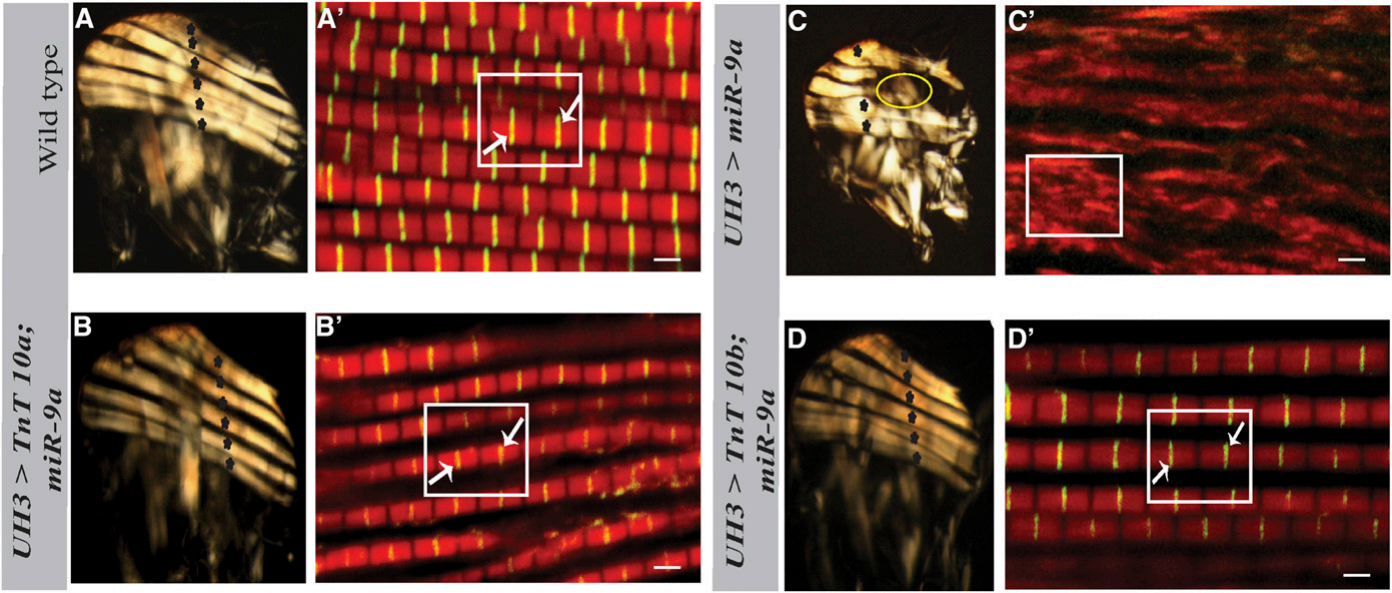
Rescue of the loss of muscle integrity. (A) Polarized light micrograph of wild-type hemithorax with six DLMs. (A’) Confocal microscopy image of wild-type muscles stained for F-actin along with Sls-GFP. The sarcomeres (box) and Z-discs (white arrows) are highlighted. (B and B’) six DLMs (asterisks) and close to normal sarcomeric structure (box) with Z-discs (white arrows) in muscles of flies expressing TnT-10a in a miR-9a overexpression background. (C and C’) Hypercontracted muscles (circled) and lack of sarcomeres (box) in muscles of flies overexpressing miR-9a. (D and D’) Six DLMs (asterisks) and restored muscle structure (box) with Z-discs (white arrows) in flies overexpressing TnT-10b in a miR-9a overexpression background (Square Box) (Bar, 2 µm). DLMs, dorsal longitudinal muscles; GFP, green fluorescent protein; miR-9a, microRna-9a.

To further confirm that that the muscle hypercontraction resulting from overexpressing miR-9a is a direct result of knockdown of TnT alone, we carried out a rescue of the muscle phenotype by overexpressing TnT devoid of the miR-9a binding site in flies with elevated levels of miR-9a in their IFMs. TnT transgenic lines were driven using *UH3-Gal4* and shifted to 29° at 50–52 hr APF. Overexpression of transgenic TnT, either the TnT 10a or 10b isoform, devoid of the miR-9a binding sequence, restored TnT protein levels in these flies compared to flies overexpressing miR-9a alone (Figure 3A). Importantly, the restoration of TnT levels in the background of miR-9a overexpression completely rescued the hypercontraction phenotype. This was evidenced by the presence of six DLMs (Figure 3B’) and the complete absence of any hypercontracted DLMs in the hemithoraces of these flies (Figure 3C). Further, the flight ability of these flies was also partially rescued: control flies [(UH3/+; +; +) Gal4 flies] were 100% up-flighted (*n* = 31); for TnT 10a, 19.6% of flies were horizontally-flighted, 28.2% down-flighted, and 52.2% flightless (*n* = 46); and for TnT 10b: 4.8% flies were up-flighted, 37.1% horizontally-flighted, 22.6% down-flighted, and 35.5% were flightless (*n* = 62); as compared to the flies with overexpression of miR-9a alone, of which were all flightless (*n* = 60) (Figure 3D). Both of these transgenic lines (TnT 10a and TnT 10b) showed almost complete restoration of muscle integrity and sarcomeric structure (Figure 4, B, B’, D, and D’) comparable to that of wild-type controls (Figure 4, A and A’), in stark contrast to the muscles in the flies overexpressing miR-9a (Figure 4, C and C’).

These data argue that TnT is the major target of the miR-9a responsible for the muscle hypercontraction phenotype. We also confirmed that the rescue of the hypercontraction phenotype by expressing TnT isoforms in IFMs is indeed because of the restoration TnT and not due to Gal4 dilution. When *UH3-Gal4* was used to drive both UAS-miR-9a and UAS-GFP, the progeny still exhibited hypercontracted muscles in hemithoraces with loss of sarcomeric structure (Figure S4, B and B’), similar to the phenotype that results from driving UAS-miR-9a alone using *UH3-Gal4* (Figure 1, D and D’).

## Discussion

The present study throws light on a new role played by miR-9a during muscle development and function. Previously, *Drosophila* miR-9a has only been shown to be involved in neuronal differentiation, wing margin patterning, and myotendinous junction formation (Biryukova *et al.* 2009; Bejarano *et al.* 2010; Yatsenko and Shcherbata 2014). We have now shown that miR-9a is involved in the translational regulation of TnT levels during sarcomeric assembly.

### TnT is a major target of miR-9a during myofibrillogenesis in the IFMs

In general, miRNAs and their targets have been observed to exhibit mutually exclusive expression (Stark *et al.* 2005). While, miR-9a is strongly expressed in all developmental stages, its expression is reduced in adult flies, including the adult IFMs where its expression is much reduced compared to earlier developmental stages (Sempere *et al.* 2003). We have confirmed that miR-9a is barely detectable in adult IFMs (Figure S1A). These data suggest that the expression of some miR-9a targets could be required for IFM development and function.

We report here that overexpression of miR-9a causes a hypercontraction phenotype in the IFMs. We identified TnT, a structural component of the thin filament of the sarcomere, as a major target for miR-9a in muscles, and have shown that miR-9a overexpression leads to repression of TnT and that this is sufficient to explain the IFM hypercontraction phenotype. In *Drosophila*, TnT is encoded by the *up* gene, is a key component in coordinating the Tn–Tm complex (Fyrberg *et al.* 1994; Nongthomba *et al.* 2007), and serves as an anchor for the other components of the complex which comprise TnI, TnC, and Tm (Gordon *et al.* 2000). Whereas the *up^1^* mutation is characterized by the absence of the adult IFM-specific TnT isoform, TnT-10a, resulting in hypercontraction (Nongthomba *et al.* 2007), the *up^101^* mutation leads to increased calcium sensitivity and irregular acto-myosin interactions, which also cause hypercontraction, producing damaged muscles (Beall and Fyrberg 1991; Nongthomba *et al.* 2003, 2007). However, previously there has been no report that epigenetic regulation of TnT can also contribute to the maintenance of stoichiometric balance. To the best of our knowledge, this is the first report on the miRNA-mediated post-translational regulation of TnT.

Clearly, downregulation of TnT by miR-9a phenocopies the mutation in *up* via the same mechanism of stoichiometric imbalance that drives the mis-regulation of the acto-myosin interaction. Importantly, this demonstrates that not only defects in transcriptional control, but also the derailing of other regulatory processes such as miRNA-mediated control, can result in the same defects. Thus, our finding that miR-9a can alter levels of thin filament components via translational control of TnT demonstrates that miRNAs are not just “regulators of regulators,” but can act as direct regulators in coordinating a complex process such as myofibrillar assembly. We show here that a major structural protein, TnT, can in fact be regulated by miRNA.

### miR-9 is required for maintaining protein stoichiometry and may have implications in the etiology of myopathies

Studies on IFM mutants indicate that structural integrity of IFMs is highly dependent on interactions between thin and thick filaments, as well as the ratio of individual myofibrillar contractile components. Any change in gene dosage and corresponding protein stoichiometry in the thin filaments translates into defects in normal myofibrillar assembly leading to hypercontraction (Tansey *et al.* 1991; Nongthomba *et al.* 2003, 2007). This explains the myofibrillar defects that result from overexpression of TnT (Marco-Ferreres *et al.* 2005), Mhc (Cripps *et al.* 1994), and in most of the heterozygotes carrying mutations in genes encoding structural proteins (Prado *et al.* 1995; Gajewski and Saul 2010).

The phenomena of both hypercontraction and hypertrophy, although observably different, are both responses to the muscle contraction and the imbalance of structural proteins. In hypercontraction, the muscles show properly arranged sarcomeres during early development, but the subsequent uncontrolled acto-myosin interactions lead to stress and muscle tearing (Nongthomba *et al.* 2003). Hypertrophy, on the other hand, is characterized by an increase in muscle volume. For instance, myocardial hypertrophy is associated with cardiac remodeling where there is an increase of muscle wall thickness, but not through any increase in myocyte number (hyperplasia). However, cardiac hypertrophy is also a physiological response to stress induced by ischemia, mitochondrial defects, and mutations in sarcomeric components, etc. Importantly, mutations in the same gene orthologs that cause hypercontraction in *Drosophila* are the ones mutated in cardiac hypertrophy patients as well. For example, mutations in the TnT gene are one of the predominant causes of hypertrophy (Seidman and Seidman 2001; Di Pasquale *et al.* 2012). Most of these TnT mutations exhibit increased calcium sensitivity and activation of muscle contractility (Harada and Potter 2004; Parvatiyar and Pinto 2015; Gilda *et al.* 2016), a similar pathogenesis to the hypercontraction produced by TnT mutants in *Drosophila* (Nongthomba *et al.* 2003, 2007). Viswanathan *et al.* (2014) have shown that the *up^101^* mutation generates a muscle abnormality similar to human cardiomyopathy through sensitive calcium regulation in the *Drosophila* heart.

Vertebrate TnT (TNNT) has a vital role in the anchoring of Tn–Tm to Actin and is also essential for Ca^+2^-mediated activation and inhibition of acto-myosin activity during muscle contraction (Potter *et al.* 1995; Schiaffino and Reggiani 1996; Domingo *et al.* 1998; Perry 1998; Oliveira *et al.* 2000). Mutations in *TNNT* in *Caenorhabditis elegans* result in defects in embryonic body wall muscle contractions and sarcomere organization (Myers *et al.* 1996). Mutations in the cardiac isoform of TnT (TNNT2) are associated with familial HCM, dilated cardiomyopathy, or arthrogryposis (Thierfelder *et al.* 1994; Kamisago *et al.* 2000; Sehnert *et al.* 2002; Sung *et al.* 2003). TNNT2 was also found to be upregulated in cardiac hypertrophic or myocardial infarction conditions (Salic and De Windt 2012). However, there are still unanswered questions pertaining to the mechanisms by which cardiac TNNT2 upregulation is brought about during hypertrophy. Hence, the upstream players that regulate the level of cardiac TNNT2 during muscle development and function are very important.

*Drosophila* miR-9a is identical to human miR-9 and the human TnT (TNNT) has significant homology to *Drosophila* TnT (Lagos-Quintana *et al.* 2001). Since miR-9a is capable of regulating TnT levels in *Drosophila*, it is possible that the human miR-9 may also play a role in regulating TNNT levels. Interestingly, sequence analysis of the human skeletal and cardiac isoforms of TNNT reveals that only the cardiac TNNT possesses the miR-9 binding site (Figure S4C) while the skeletal isoforms lack it. Incidentally, bioinformatic analysis failed to find a miR-9 target site in the mRNA sequence of mouse TNNT. It is important to note that the initial report of miR-9’s role in muscle hypertrophy were from studies on mice (Wang *et al.* 2010), so miR-9a could be playing varied roles in different organisms. Our study suggests that miR-9a might be involved in specifically regulating the levels of cardiac TnT in humans. It would be interesting to know if the increase in cardiac TNNT that occurs in response to hypertrophic stimulus is mediated by miR-9. Many mutations of the human cardiac TnT give rise to the hypertrophic condition (Di Pasquale *et al.* 2012). However, cardiac hypertrophy is a genetically and clinically heterogeneous disorder and its etiology in many instances has not been determined (Gilda *et al.* 2016). The present study provides a plausible candidate in the form of miR-9 to explore in the etiology of idiopathic cardiomyopathies. It would be interesting to determine what represses miR-9 during myofibril assembly. Its continuous expression would be deleterious to myofibril assembly through its repression of the expression of very important structural proteins such as TnT. Taurine-upregulated gene-1 (TUG1) negatively regulates miR-9 in a human cancer cell line (Zhao and Ren 2016). Whether TUG1 or similar protein(s) are involved in muscle hypertrophy/hypercontraction and myofibril assembly requires further investigation.

## 

## Supplementary Material

Supplemental material is available online at www.g3journal.org/lookup/suppl/doi:10.1534/g3.117.300232/-/DC1.
