# Supplementary figures and images for: Overexpression of miRNA-9 Generates Muscle Hypercontraction Through Translational Repression of Troponin-T in Drosophila melanogaster Indirect Flight Muscles
Source: G3 (Bethesda). 2017 Sep 1;7(10):3521–31. doi: 10.1534/g3.117.300232 (PMC5633399; doi:10.1534/g3.117.300232)

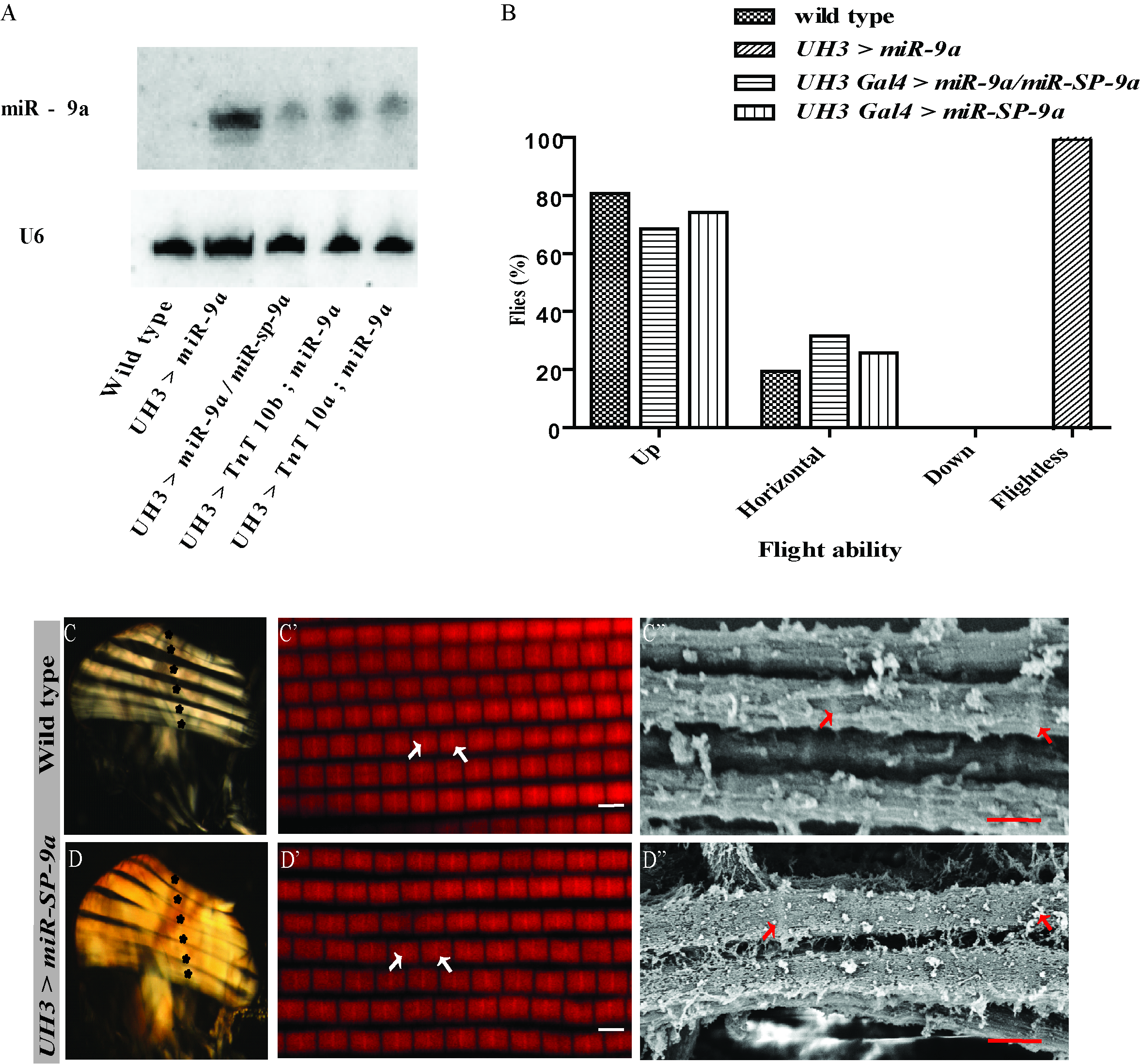

Supplement: Supplementary file 1 [file 3521FigS1.tif]

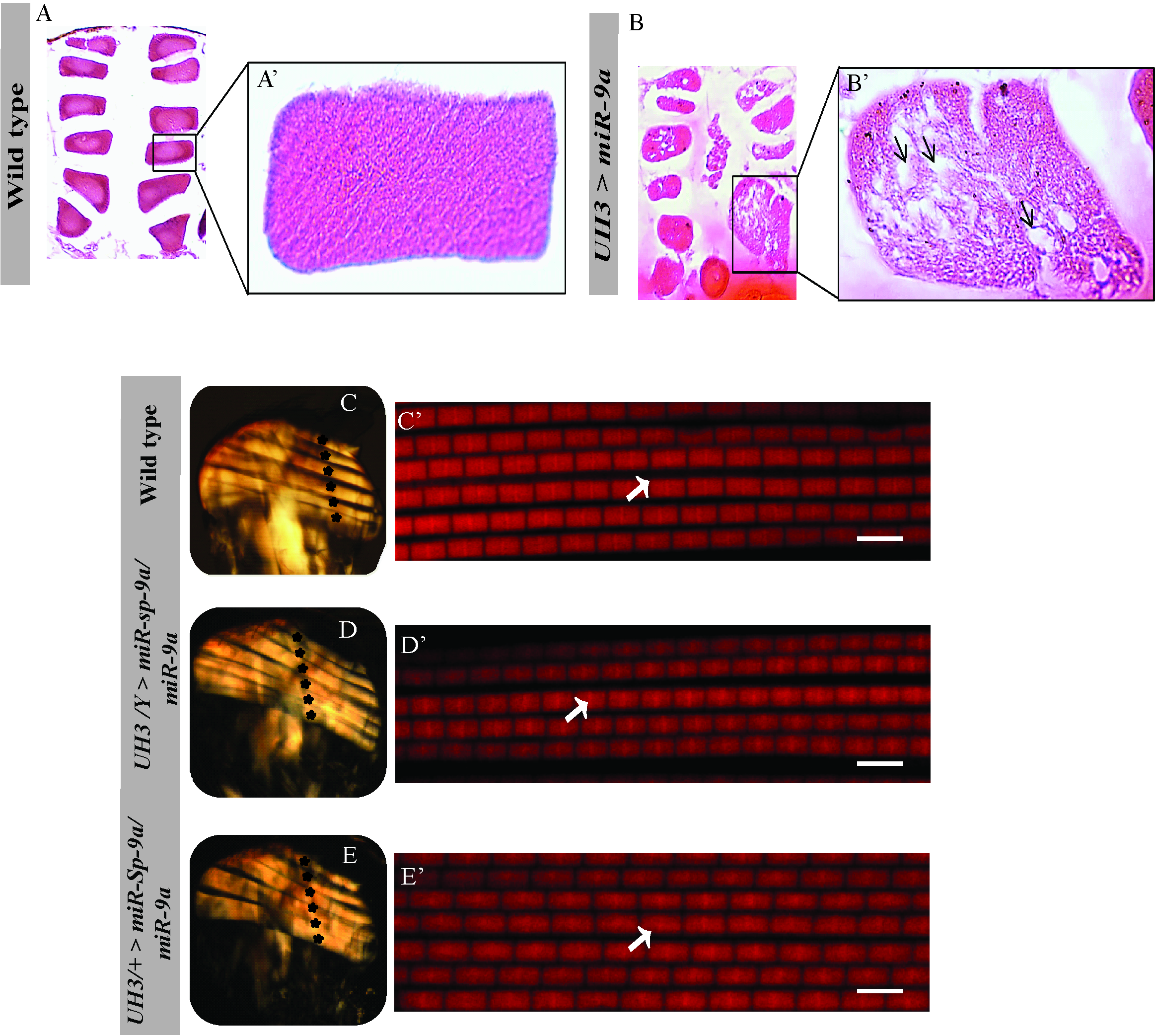

Supplement: Supplementary file 2 [file 3521FigS2.tif]

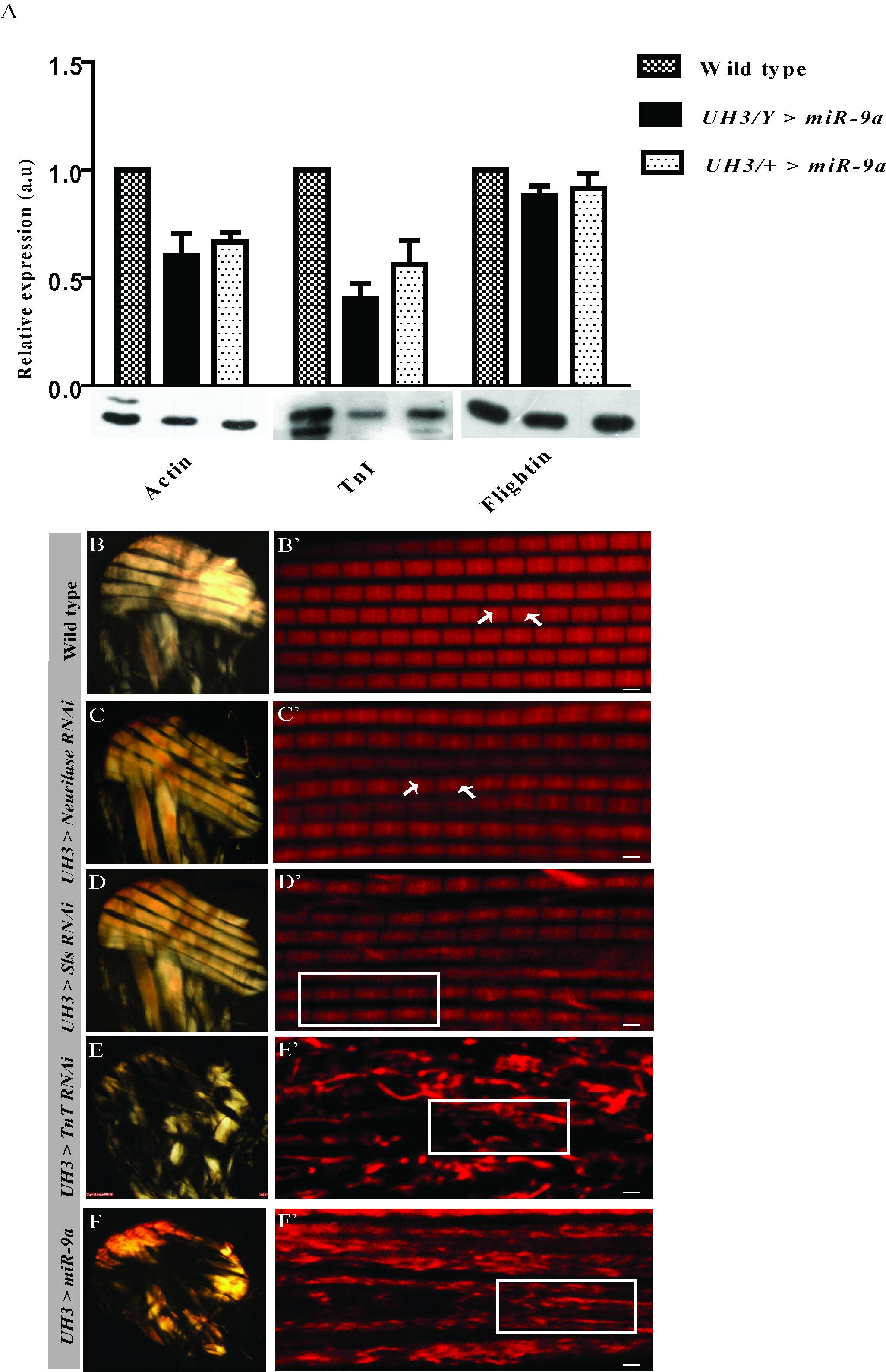

Supplement: Supplementary file 3 [file 3521FigS3.tif]

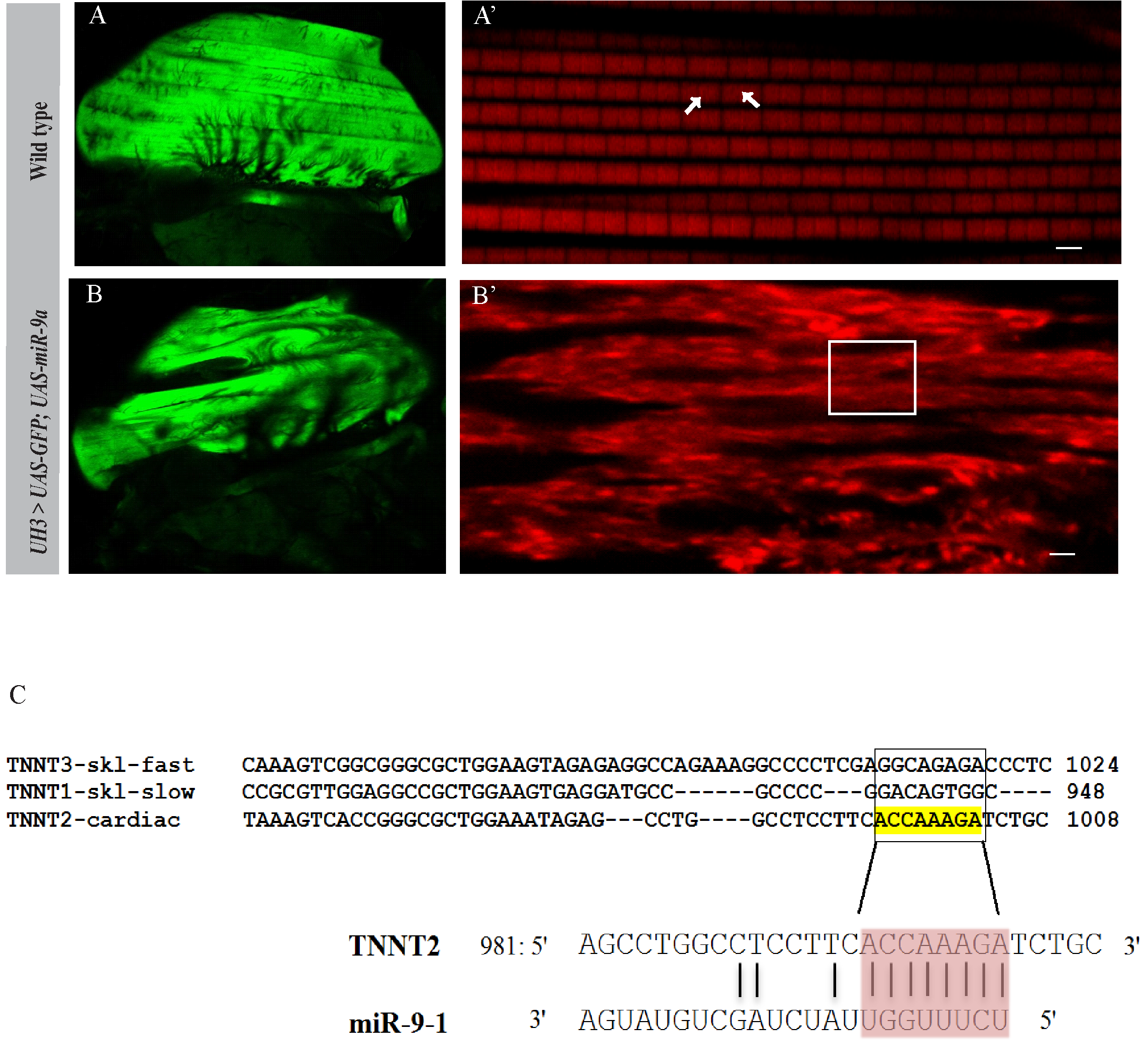

Supplement: Supplementary file 4 [file 3521FigS4.tif]
