# Supplementary material for: Overexpression of miRNA-9 Generates Muscle Hypercontraction Through Translational Repression of Troponin-T in Drosophila melanogaster Indirect Flight Muscles
Source: G3 (Bethesda). 2017 Sep 1;7(10):3521–31. doi: 10.1534/g3.117.300232 (PMC5633399; doi:10.1534/g3.117.300232)
Supplement: Supplementary file 5 [file 3521FileS5.docx]

**Figure Legends**

**Fig. 1: IFM-specific over-expression of miR-9a causes muscle hypercontraction.** (A) Wild-type adult flies with normal wing posture. (A’) Regular wing position along the body axis in a wild type fly. (B) Adult flies over-expressing miR-9a have upheld wings. (B’) Upright wing phenotype in a fly over-expressing mir-9a. (C) Polarized light micrograph of dissected wild-type adult hemi-thorax showing six DLMs (asterisks). (C’-C’’) Adult myofibrils stained with Phalloidin-TRITC (F-actin) and Sls-GFP (arrows indicate Z- disc). (C’’’) SEM micrograph of wild-type myofibril (arrow indicates Z - disc). (D) Polarized light image of hemi-thorax of adult male with over-expression of miR-9a showing broken muscles (arrow) and clumped muscle (outlined in black and orange). (D’-D’’) Loss of sarcomeric structural integrity (box) in flies over - expressing miR-9a. (D’’’) SEM micrograph showing absence of proper sarcomeres (box) after miR-9a over-expression (Scale bar 2 µm). (E) Flight assay of the miR-9a over-expression flies. (F) Images of wild-type hemi-thorax showing six DLMs (asterisks) under polarized light. (F’) Phalloidin-TRITC stained myofibril showing normal sarcomeric structure (arrow) in wild type flies. (G) Adult flies carrying a myosin *Mhc^P401S^* mutation in the over-expression of miR-9a background show six DLMs (asterisks) in the hemi-thorax and (G’) normal sarcomeres (arrows) in the myofibrils. (H) Polarized light image of hemi-thorax from flies with over-expression of miR-9a. (H’) Abrogated sarcomeric structure (box) in the myofibrils of flies over-expressing miR-9a (Scale bar 2 µm).

**Fig. 2: Putative target genes of miR-9a that are involved in muscle development.** (A) Lists of genes which are putative targets of miR-9a and their functions in muscles. The upheld gene that encodes Troponin T is highlighted in red (within blue box) (B) Expression profile of the putative miR-9a targets in the IFMs obtained from the microarray data from IFM of wild type flies. The *upheld* gene showed highest expression in IFM (highlighted by the blue bar). (C) Relative expression of the target genes validated by real-time PCR. (D) Schematic representation of miR-9a binding site at 3’-UTR of upheld (TnT) and the mechanism of translation repression of Troponin T. (E) Quantification of the relative expression of TnT after miR-9a over-expression, using *α*-Tubulin as loading control.

**Fig. 3: Transgenic lines with over-expression of TnT (10a or 10b isoform) restore Troponin-T levels and rescue the muscle hypercontraction phenotype resulting from over-expression of miR- 9a.** (A) Western blots and the quantification of the relative expression of Troponin-T (loading control *α*-Tubulin). (B) Polarized light micrograph showing normal six DLMs following the over-expression of TnT-10a or (B’) TnT-10b isoforms in the background of miR-9a over-expression. (B’’) Polarized image showing hypercontracted muscles after miR-9a over-expression. (C) Quantification of the percentage of flies over-expressing miR-9a that present with hypercontraction phenotype after restoration of TnT levels. (D) Flight data for the flies over-expressing TnT 10a or 10b isoforms in the background of over-expression of miR- 9a.

**Fig. 4: Rescue of the loss of muscle integrity.** (A) Polarized light micrograph of wild type hemi-thorax with six DLMs. (A’) Confocal microscopy image of wild type muscles stained for F-actin along with Sls-GFP. The sarcomeres (box) and Z- discs (white arrows) are highlighted. (B-B’) 6 DLMs (asterisks) and close to normal sarcomeric structure (box) with Z- discs (white arrows) in muscles of flies expressing TnT-10a in mi-9a over-expression background. (C-C’) Hypercontracted muscles (circled) and lack of sarcomeres (box) in muscles of flies over-expressing miR-9a. (D-D’) Six DLMs (asterisks) and restored muscle structure (box) with Z- discs (white arrows) in flies over-expressing TnT-10b in miR-9a over-expression background (Square Box). (Scale bar 2 µm).

**Supplementary figure legends**

**Fig. S1:** **miR-9a depletion did not affect normal muscle structure and function.** (A) Northern blot analysis of miR-9a expression in IFM of various genotypes. (B) Flight data following knockdown of miR-9a and knockdown of miR-9a in the over-expression background. (C) Polarized light image of hemi-thorax of adult wild type flies showing six DLMs (asterisks). (C’) Confocal images of wild type myofibrils stained with Ph-TRITC (F-actin) showing normal sarcomeres and Z- discs (arrows) (C’’) SEM micrograph of wild-type myofibril. Z- discs are highlighted (red arrows). (D) Adult hemi-thorax of a fly with miR-9a depletion shows 6 DLMs (asterisks). (D’) Ph-TRITC stained muscles of flies with knockdown of miR-9a show normal sarcomeric structure (arrow). (D’’) SEM micrographs of myofibrils from IFM of flies with miR-9a depletion (C’-D’, scale bar 2 μm).

**Fig. S2:** (A) H & E staining of transverse sections (TS) of wild-type hemi-thorax. (A’) Single DLM from the TS of wild type hemi-thorax. (B) H & E staining of hemi-thorax of a fly with miR-9a over expression. (B’) TS of a single DLM in flies over-expressing miR-9a showing disintegration of muscle structure (Black arrows). ). (C) Polarized light images of hemi-thorax of adult wild type flies show six DLMs (asterisks). (C’) Confocal images of wild-type myofibrils with normal sarcomeric integrity (arrow). (D) Adult hemi-thorax of a male fly with miR-9a depletion in the miR-9a over-expression background shows 6 DLMs (asterisks). (D’) Ph-TRITC stained myofibrils of male flies with knockdown of miR-9a in the over-expression background show normal sarcomere structure (arrow). (E) Hemi-thorax of a female fly with knockdown of miR-9a in the over-expression background showing 6 normal DLMs (asterisks). (E’) Myofibrils from female flies with miR-9a depletion in the miR-9a over-expression background with normal sarcomeric structure (arrow). Scale bar 2 μm.

**Fig. S3:** **miR-9a knock down under miR-9a over expression rescues muscle structure and function.** (A) Western blots and the quantification of the relative expression of thin filament proteins (Actin and TnI) and thick filament protein (Flightin). (B) Adult wild-type hemi-thorax of a fly showing 6 DLMs (asterisks). (B’) Confocal images of wild type myofibrils stained with Ph-TRITC showing normal sarcomeric structure (white arrows). (C) Flies with knock down of *neuralized* show 6 DLMs (asterisks) in the hemi-thorax and (C’) show intact sarcomere structures (white arrows) in myofibrils. (E) Adult flies with knockdown of *Salimus (Sls)* show six DLMs and (E’) slightly irregular sarcomeres respectively (rectangle). (F) Flies with knock down of Troponin-T (*upheld*) show hypercontracted muscles and (F’) complete absence of sarcomeres (box). (G) Hemi-thorax of a fly with miR-9a over-expression shows hypercontracted muscles and (G’) absence of sarcomeres (box). Scale bar 2 μm.

**Fig. S4:** (A) Presence of normal 6 DLMs in the wild type hemi-thorax where UAS-GFP is driven by *UH3-Gal4*. (A’) Well-arranged sarcomeric structure (white arrows) in the myofibrils of wild type IFMs. (B) Hemi-thoraxes of flies with *UH3-Gal4* driven over-expression of miR-9a along with UAS-GFP showed hypercontracted muscles and (B’) complete loss of sarcomere structures (box). Scale bar 2 μm. (C) Only the human cardiac muscle-specific Troponin T isoform (TnnT 2) has a binding site for human miR-9.
